# Immuno-instructive biomaterials for coronary artery disease and myocardial infarction repair

**DOI:** 10.1007/s10856-026-07096-1

**Published:** 2026-06-25

**Authors:** Xuanfu Chi, Chaowei Wu, Feifei Ni, Tai Zeng, Songsong Wu, Yunrui Zhang

**Affiliations:** 1 Alberta institute, Wenzhou Medical University, Wenzhou, China; 2 School of Ophthalmology and Optometry, Wenzhou Medical University, Wenzhou, China; 3 The Second Clinical Medical College, Wenzhou Medical University, Wenzhou, China; 4 Department of Blood Transfusion Medicine, Heyuan People’s Hospital, Guangdong Provincial People’s Hospital Heyuan Hospital, Heyuan, China; 5 Department of Radiation Oncology, the Third Affiliated Hospital of Wenzhou Medical University, Wenzhou, China; 6 Department of Cardiology, the Third Affiliated Hospital of Wenzhou Medical University, Wenzhou, China

**Keywords:** Coronary artery disease, Myocardial infarction, Biomaterials, Immunomodulation, Tissue repair

## Abstract

Coronary artery disease (CAD) and myocardial infarction (MI) feature a highly dynamic inflammatory milieu. While reperfusion remains essential, it does not sufficiently correct immune dysregulation or prevent adverse remodeling. Biomaterials offer a complementary strategy by enabling spatiotemporally controlled immunomodulation and tissue repair. By providing localized delivery of bioactive cues, mechanical support to stabilize the infarct wall, and immuno-instructive interfaces that steer macrophages toward reparative phenotypes, biomaterial platforms can reshape the post-infarction microenvironment. This review summarizes recent biomaterial-mediated strategies for the treatment of coronary heart disease. By integrating advances in immunomodulatory design, controlled drug delivery, and cardiac tissue engineering, it highlights how biomaterials can reshape the post-ischemic microenvironment to improve repair and limit adverse remodeling. These insights not only clarify current opportunities and translational barriers, but also provide guidance for developing next-generation, phase-adaptive and clinically scalable biomaterial platforms.

**Figure:**
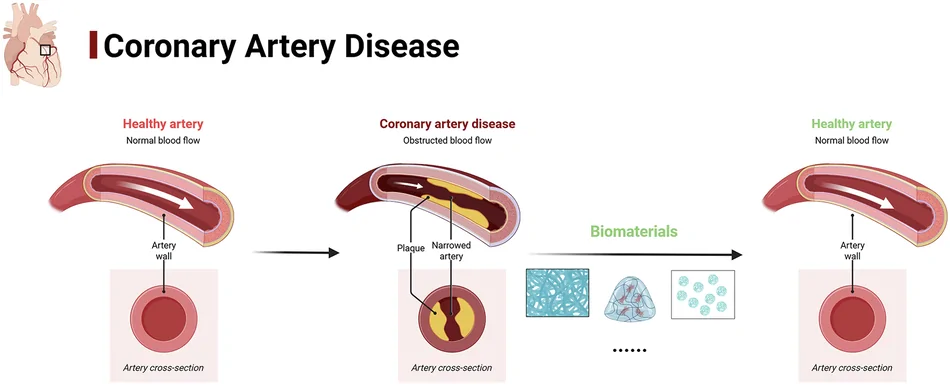

## Introduction

Coronary artery disease (CAD), driven primarily by coronary atherosclerosis, remains a leading cause of morbidity and mortality worldwide [1]. Beyond luminal stenosis and acute thrombotic occlusion, CAD progression and adverse outcomes are strongly shaped by a dynamic inflammatory milieu that spans plaque initiation [2], destabilization, myocardial infarction (MI) [3], and post-MI remodeling [4]. Although timely reperfusion by percutaneous coronary intervention (PCI) or thrombolysis is indispensable for acute MI [5], reperfusion injury, limited cardiomyocyte regenerative capacity, and maladaptive immune responses frequently culminate in infarct expansion, scar formation, left ventricular (LV) dilation [6], and heart failure [7]. Emerging biomaterial-based therapies offer a complementary paradigm that extends beyond restoring blood flow, aiming to modulate local inflammation, stabilize extracellular matrix (ECM) turnover [8], enhance neovascularization, and enable regenerative signaling with spatiotemporal precision. Here, we summarize (i) the inflammatory basis of CAD and post-MI remodeling [9], (ii) the rationale and functional modalities of biomaterials for immunomodulation and tissue repair, and (iii) microenvironment-centered strategies targeting macrophage polarization, endothelial regeneration, and cardiomyocyte proliferation [10]. We further discuss translational barriers and propose design principles for next-generation immuno-instructive biomaterials for CAD and MI.

## Research background

**Fig. 1:**
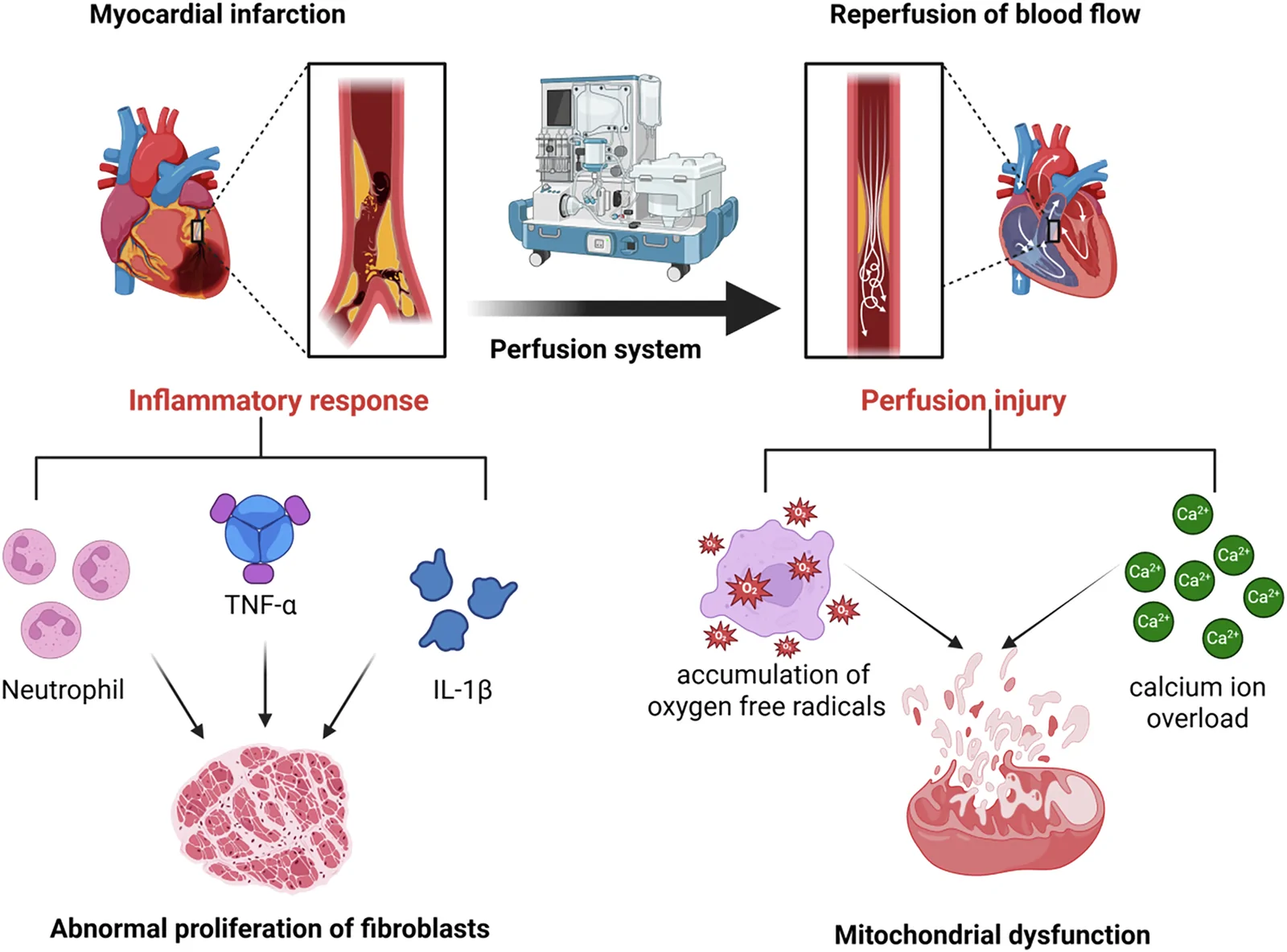
The principle of myocardial infarction and clinical perfusion injury

Coronary artery disease (CAD), also referred to as coronary atherosclerotic heart disease, is an ischemic cardiac disorder fundamentally caused by atherosclerotic lesions in the coronary arteries [11]. Lipid deposition, fibrous hyperplasia, and vascular calcification progressively narrow the arterial lumen or precipitate acute occlusion, resulting in myocardial ischemia and hypoxia and, in severe cases, irreversible cardiomyocyte necrosis. CAD may involve a single or multiple coronary vessels [12], and clinical severity is determined by the degree of stenosis as well as superimposed acute events such as thrombosis and vasospasm (Fig. 1).

According to ischemic burden, clinical manifestations, and disease course, CAD is commonly categorized into: (i) silent (asymptomatic) CAD, characterized by significant coronary stenosis without typical symptoms at rest but detectable ischemia on stress testing; (ii) chronic coronary syndromes, dominated by stable [13], long-standing ischemia, including stable angina triggered by exertion or emotional stress and ischemic cardiomyopathy due to prolonged hypoperfusion; and (iii) acute coronary syndromes (ACS), the high-risk spectrum driven by unstable plaque disruption and superimposed thrombosis, encompassing unstable angina, non–ST-segment elevation myocardial infarction (NSTEMI) [14], and ST-segment elevation myocardial infarction (STEMI) [15].

The pathogenic cascade of CAD begins with endothelial dysfunction induced by risk factors such as hypertension and dyslipidemia. Low-density lipoprotein infiltration into the subendothelial space and subsequent oxidative modification initiate sterile inflammation, recruiting circulating monocytes that differentiate into macrophages and internalize lipids to form foam cells [16]. Together with proliferating vascular smooth muscle cells and collagen deposition, these components constitute the atherosclerotic plaque [17]. As lesions advance, stable plaques typically cause chronic luminal narrowing, whereas vulnerable plaques are prone to rupture, activating platelet aggregation and thrombus formation. Complete coronary occlusion results in STEMI, in which sustained ischemia drives cardiomyocyte death.

Following MI, damaged myocardium releases danger-associated molecular patterns (DAMPs) that activate innate immunity [18]. The post-infarction response is often conceptualized as sequential yet overlapping phases: an inflammatory phase dominated by neutrophils and classically activated (M1-like) macrophages that clear necrotic debris; a proliferative phase in which alternatively activated (M2-like) macrophages promote angiogenesis and matrix deposition [19]; and a maturation phase characterized by fibroblast-driven scar formation that stabilizes the ventricular wall but lacks contractility. When immune resolution fails—manifested by excessive inflammation, uncontrolled ECM degradation [20], or impaired reparative signaling—adverse outcomes ensue, including infarct expansion, LV remodeling (dilation and geometric distortion), and progression to heart failure.

The clinical burden of CAD is substantial due to its high prevalence and high rates of death and disability. STEMI carries particularly high short-term mortality, and even after successful reperfusion, patients remain vulnerable to lethal complications [21]. Critically, adult cardiomyocytes exhibit limited regenerative capacity; thus, infarcted myocardium is largely replaced by fibrotic scar tissue, leading to persistent loss of systolic function.

Immune microenvironment regulation represents a major opportunity to improve outcomes in CAD and MI by correcting the imbalance between tissue injury and repair. Inflammation underlies early plaque formation (lipid oxidation–driven chronic inflammation), drives plaque destabilization, and shapes post-MI remodeling [22]. In acute MI, DAMP-triggered innate immune activation recruits neutrophils and pro-inflammatory macrophages that release cytokines (e.g., TNF-α, IL-1β) and proteases (e.g., matrix metalloproteinases, MMPs) [23]. While necessary for debris clearance, excessive or prolonged activation amplifies cardiomyocyte apoptosis, accelerates ECM degradation, and enlarges infarct size. Conversely, inadequate transition toward reparative programs (e.g., impaired M1-to-M2 polarization, dysregulated IL-10/TGF-β signaling) compromises neovascularization and promotes maladaptive fibrosis, thereby accelerating LV remodeling and heart failure. Accordingly, restoring immune homeostasis can (i) reduce acute injury by restraining excessive inflammation (e.g., NLRP3 inflammasome or IL-1β blockade), (ii) enhance reparative angiogenesis and granulation by promoting pro-resolving macrophage programs and growth factor production [24] (e.g., VEGF, PDGF), and (iii) stabilize ECM dynamics by rebalancing MMPs and their endogenous inhibitors. This immunological perspective complements conventional interventions (revascularization, antiplatelet therapy) by directly targeting the inflammatory cascade that drives long-term deterioration.

## Therapeutic roles of biomaterials in MI and CAD

Current MI management prioritizes rapid reperfusion to salvage jeopardized myocardium [25], typically via emergency PCI or intravenous thrombolysis. However, this approach has intrinsic limitations. Reperfusion can induce “reperfusion injury,” [26, 27] in which abrupt oxygen reintroduction triggers reactive oxygen species (ROS) bursts and calcium overload, leading to mitochondrial dysfunction and energetic collapse, thereby exacerbating cardiomyocyte apoptosis [28]. Moreover, because adult cardiomyocytes have minimal capacity for self-renewal, infarcted tissue is largely replaced by fibrotic scar, predisposing to progressive LV remodeling and functional decline.

A key bottleneck across these processes is post-MI immune dysregulation: excessive pro-inflammatory activation, impaired resolution, and disrupted reparative signaling collectively impede effective healing. In this context, biomaterials have emerged as a versatile platform to modulate inflammation and promote myocardial repair through coordinated, multi-functional mechanisms.

### Spatiotemporally controlled delivery of therapeutics

Biomaterials with favorable biocompatibility and biodegradability can serve as local depots for anti-inflammatory cytokines (e.g., IL-10, TGF-β), M2-polarizing cues, or MMP modulators, prolonging retention within the infarct zone while minimizing systemic exposure. For example, injectable hydrogels can buffer excessive pro-inflammatory signaling and reduce neutrophil infiltration by sustained local release of anti-inflammatory peptides or proteins [29].

### Mechanical stabilization and ECM substitution

Cardiac patches and supportive scaffolds can partially substitute for damaged ECM and reduce pathological wall stress, indirectly attenuating inflammatory signaling and limiting mechanically driven secondary injury [30]. By providing physical guidance, such constructs may also facilitate ordered tissue repair and cellular integration.

### Immuno-instruction and macrophage reprogramming

Material architecture, surface chemistry, and charge can bias immune cell recruitment and polarization. Porous structures can facilitate macrophage infiltration and favor reparative phenotypes that secrete pro-angiogenic and pro-regenerative factors (e.g., VEGF, PDGF), accelerating neovascularization and granulation tissue formation [31].

### Restoration of electro-mechanical coupling

Conductive biomaterials can improve electrical signal propagation and synchronize contraction [32], thereby reducing arrhythmia-related stress and potentially dampening inflammation secondary to electrical dysfunction.

Representative studies illustrate these concepts. Early porous, mechanically compliant scaffolds attempted to recapitulate myocardial mechanics and support cell engraftment [33]. More recent adhesive bioresorbable and conductive patches integrate mechanical support with electrical conductivity and immunomodulatory interfaces that can promote reparative macrophage responses [34, 35]. Injectable hydrogels offer minimally invasive delivery and conformal filling of irregular infarct cavities, enabling sustained release of therapeutics and pH/ROS-responsive microenvironment conditioning [36]. Collectively, these designs converge on a shared objective: interrupting the “injury–inflammation–remodeling” loop by combining physical shielding, chemical signaling, and biological activation of endogenous repair.

Despite promising preclinical efficacy, clinical translation remains constrained by surgical invasiveness for patch implantation, challenges in co-optimizing conductivity and bioactivity, and long-term safety evaluation of degradation products. Nevertheless, biomaterials provide a distinct therapeutic dimension beyond “reperfusion-centric” care, enabling immune microenvironment remodeling and functional restoration across the post-MI trajectory.

## Microenvironment-remodeling strategies for MI therapy

Immune mediators regulate the inflammatory microenvironment after MI by orchestrating immune cell recruitment and polarization, ECM turnover, angiogenesis, and scar maturation [37]. Broadly, these mediators can be conceptualized as: (i) pro-inflammatory regulators that require restraint to limit secondary injury; and (ii) anti-inflammatory/pro-reparative factors that should be augmented to promote resolution and regeneration [38]. Importantly, their therapeutic manipulation must respect the temporal sequence of post-MI healing and the spatial heterogeneity within infarct, border, and remote zones [39].

### Pro-inflammatory mediators to restrain excessive injury

#### TNF-α

Primarily produced by pro-inflammatory macrophages and neutrophils (and by stressed cardiomyocytes), TNF-α amplifies inflammatory cascades via TNFR1/2 signaling, activates NF-κB/MAPK pathways, promotes leukocyte recruitment [40], and can directly induce cardiomyocyte apoptosis. While transient early expression may aid debris clearance, excessive TNF-α contributes to infarct expansion and adverse remodeling [41]. Therapeutic inhibition can reduce inflammatory injury in experimental models; however, clinical benefits appear context- and timing-dependent, emphasizing the need for stage-specific intervention.

#### IL-1β

IL-1β is a key driver of sterile inflammation after MI, generated upon inflammasome activation and caspase-1–dependent processing [42]. It promotes leukocyte recruitment, amplifies cytokine networks, and enhances ECM degradation through MMP induction, weakening infarct wall integrity. Targeting IL-1 signaling has shown promise in reducing systemic inflammation and improving remodeling indices, supporting its value as a microenvironmental modulator, particularly in the early inflammatory phase.

#### MMPs

MMPs regulate ECM turnover but are detrimental when excessively activated, leading to ECM breakdown, infarct wall thinning, and LV dilation [43]. Broad-spectrum inhibition has been limited by off-target effects and impaired healing, motivating the development of more selective strategies (e.g., targeted MMP-9 modulation) and biomaterial-enabled local control to preserve necessary remodeling while preventing pathological degradation [44].

### Anti-inflammatory and reparative mediators to promote healing

#### IL-10

IL-10 is a central pro-resolving cytokine produced by reparative macrophages, regulatory T cells, and B cells [45]. It suppresses pro-inflammatory transcriptional programs and supports macrophage polarization toward tissue-repair phenotypes. Local, sustained delivery (e.g., hydrogel depots or nanoparticles) can accelerate inflammatory resolution while minimizing systemic immunosuppression [46].

#### TGF-β

TGF-β exerts dual roles: it supports granulation tissue formation, fibroblast activation, and vascular stabilization in the proliferative phase [47], yet excessive or prolonged signaling promotes maladaptive fibrosis and ventricular stiffening during maturation. Therefore, time-programmed regulation—enhancing early reparative activity while preventing late overactivation—is critical [48], and biomaterials are particularly suited for staged release or switchable inhibition.

#### VEGF and PDGF

VEGF is pivotal for endothelial proliferation and neovessel formation, whereas PDGF contributes to vessel maturation and mural cell recruitment [49]. Their coordinated signaling supports both the establishment and stabilization of new vasculature. Biomaterials can mitigate limitations of bolus protein delivery (short half-life, poor retention, systemic leakage) by enabling controlled, localized release and by presenting matrix cues that improve vascular organization [50].

#### IL-4 and IL-13

These cytokines promote alternative macrophage activation and support resolution of inflammation [51]. When delivered in a controlled manner, they can accelerate the transition toward reparative immune states, though dosing and timing must be managed to avoid excessive fibrosis.

### Summary of immune-factor–guided therapy

Post-MI immune modulation should follow phase-specific objectives: restrain excessive pro-inflammatory mediators early to limit secondary injury; enhance pro-resolving and pro-angiogenic factors during proliferation to drive repair; and balance fibrotic cues during maturation to prevent pathological scarring and stiffness. Therapeutic development is shifting from single-factor administration toward multi-factor co-delivery, factor–material conjugation, and integration with cell-based approaches, guided increasingly by single-cell profiling of immune and stromal subsets to enable precision microenvironment control Tables 1–3.

**Table 1:** Biological factors involved in myocardial infarction

Inflammatory effect	Factor	Source	Function	The role in myocardial infarction	Therapeutic significance	References
Inflammatory	**TNF-α**	M1 type macrophages	By activating the TNFR1/2 receptors and triggering the NF-κB and MAPK signaling pathways, it induces the transcription of downstream pro-inflammatory factors (IL-1β, IL-6)	Early moderate expression can promote the clearance of necrotic tissue, but excessive release will lead to secondary apoptosis of myocardial cells and expansion of the infarcted area.	Inhibiting the activity of TNF-α can reduce myocardial cell apoptosis and the spread of inflammation.	[41, 81, 82]
		Neutrophil	Promote neutrophil infiltration			
		Cardiomyocytes	Directly activate the apoptotic pathway of cardiac muscle cells (such as caspase-8)			
	**IL-1β**	M1 type macrophages	By activating the MyD88-dependent signaling pathway through the IL-1R1 receptor	IL-1β is the key mediator of asymptomatic inflammation, and its level is positively correlated with the infarct size after myocardial infarction and the risk of malignant arrhythmia.	The recombinant human IL-1 receptor antagonist has shown in small-scale clinical trials the ability to reduce the level of hs-CRP after myocardial infarction and improve left ventricular ejection fraction.	[83, 84]
			Induction of pro-inflammatory factors (TNF-α, IL-6) release			
		Dendritic cells	Promote neutrophil chemotaxis; simultaneously enhance the expression of MMPs			
			Degradation of collagen fibers, weakening the wall strength of the infarcted area			
	**MMPs**	M1 type macrophages	Degradation of extracellular matrix (ECM) components	During the inflammatory phase, excessive expression of MMPs leads to the disintegration of the ECM in the infarcted area, resulting in thinning of the ventricular wall, dilation of the ventricular cavity, and promotion of the expansion of the infarcted area to the surrounding myocardium.	Reduce the expansion of the infarcted area	[85]
		Neutrophil	Activate pro-IL-1β and amplify the inflammatory signal		Inhibiting the activity of MMPs can alleviate matrix degradation and remodeling.	
		Fibroblast				
**Anti-inflammatory**	**IL-10**	M2 type macrophages	Activate the JAK1/STAT3 pathway through the IL-10R receptor and inhibit the transcriptional activity of NF-κB	IL-10 gene therapy can reduce the proportion of M1 macrophages in the infarcted area and increase the proportion of M2 macrophages.	Exogenous IL-10 delivery can increase the concentration of IL-10 in the targeted area.	[86, 87]
		Regulatory T cells	Reduce the release of pro-inflammatory factors such as TNF-α and IL-1β			
		B cells secrete	At the same time, it promotes the polarization of M2 macrophages and inhibits the infiltration of neutrophils.			
	**TGF-β**	M2 type macrophages	Promote fibroblast proliferation and differentiation	Moderate expression during the proliferative phase can promote the formation of granulation tissue, but excessive activation during the mature phase will lead to excessive collagen deposition, increased myocardial stiffness, and accelerate the progression to congestive heart failure.	During the proliferative phase, TGF-β expression is promoted to accelerate repair. In the mature phase, its excessive activation is inhibited to prevent fibrosis.	[88–90]
		Fibroblast	Inhibit T cell proliferation and release of pro-inflammatory factors			
		Platelets	Promote the recruitment of pericytes and stabilize the newly formed blood vessels			
	**VEGF**	M2 macrophages	By activating the PI3K/Akt/eNOS pathway through the VEGFR2 receptor, it promotes the proliferation, migration and lumen formation (angiogenesis) of endothelial cells.	The level of VEGF during the proliferation phase determines the efficiency of angiogenesis.	VEGF delivery is a core strategy for promoting angiogenesis. In clinical applications, bio-materials are used to load VEGF in order to increase the VEGF level in the local area.	[50, 91, 92]
		Endothelial cells

**Table 2:** Drug system for treating myocardial infarction

Biological materials	Active factor	Delivery route	Ref
Enzyme-Responsive Nanoparticles	MMPi	NPs	[93]
Nanoparticle platform with a low glutathione (GSH) response	PF543		[94]
Delivery of liquid metal particles and tanshinone IIA	Liquid metal (LMs), tanshinone IIA (TA) drug	Sodium alginate (SA) solution	[32]
Targeted miR-21 loaded liposomes	MIR21	Cardiac troponin T	[95]
Stimulus-Responsive Multifunctional Hydrogels	Curcumin (Cur)	collagen type III (rhCol III) is loaded in the hydrogel	[96]
Mimetic Peptide and Mitochondria-Targeted Antioxidant-Loaded Hydrogel System	QK&SS31	self-assembled nanofibrous hydrogels	[97]

**Table 3:** Clinical trial progress of biomaterials in the field of myocardial infarction

Biomaterial/produ-ct	Delivered cargo or functional component	Route of administrat-ion	Clinical phase/status	Main enrolled population	Primary endpoints/outcome measures	Results and clinical progress	Registr-ation number
VentriGel, porcine decellularized myocardial extracellular matrix hydrogel	Primarily delivers a bioactive myocardial ECM scaffold, rather than an exogenous drug, to provide a pro-reparative microenvironment	Catheter-based transendocardial injection using the NOGA/Myostar system	Phase I, open-label, completed	Patients with prior large STEMI after PCI-mediated revascularization and reduced left ventricular function; reported enrollment window from 60 days to 3 years after MI, with LVEF approximately 25–45%	Primary endpoints focused on safety, including adverse events and laboratory parameters; secondary outcomes included CMR-based assessment, LVEF, LVESV, LVEDV, infarct size, 6-min walk test, NYHA class, and BNP levels	A 15-patient study showed that catheter-based injection was safe and feasible, with no product-related serious safety concerns reported. Improvements in functional indicators such as 6-min walk distance and NYHA class were observed, but the study was small and non-randomized	NCT02305602
IK-5001 / BL-1040 / Bioabsorbable Cardiac Matrix (BCM), alginate hydrogel	No exogenous drug cargo; functions as a bioresorbable ECM-mimetic or mechanical support material intended to attenuate post-MI left ventricular dilatation	Intracoronary injection through the infarct-related coronary artery after acute STEMI, followed by in situ hydrogel formation	First-in-human pilot study and multicenter randomized trial; NCT01226563 registered	Patients with acute STEMI after successful PCI and stent implantation	Safety, left ventricular remodeling, LVEDV, LVESV, and heart failure-related clinical events	Early first-in-human data suggested that intracoronary delivery was feasible and generally well tolerated. However, subsequent larger randomized studies did not demonstrate clear improvement in left ventricular remodeling or clinical outcomes, limiting further translational development	NCT01226563
Algisyl-LVR, alginate hydrogel	No drug cargo; serves as a long-term intramyocardial mechanical bulking and support material to reduce left ventricular wall stress	Multiple intramyocardial injections into the left ventricular wall via a minimally invasive surgical or open-chest approach	Pilot/Phase II studies; AUGMENT-HF randomized controlled trial completed	Patients with advanced chronic heart failure, including ischemic and non-ischemic dilated cardiomyopathy, often associated with post-MI ventricular remodeling	In AUGMENT-HF, the primary endpoint was peak VO₂ at 6 months; secondary endpoints included 6-min walk distance, NYHA class, KCCQ score, and safety outcomes	In a 78-patient randomized study, Algisyl-LVR plus standard medical therapy improved exercise capacity and symptoms compared with standard therapy alone. However, this was primarily a heart failure/ventricular remodeling study rather than an acute MI treatment trial; perioperative risk and surgical invasiveness remain important translational concerns	NCT01311791
XDROP / catheter-delivered alginate hydrogel system, high-stiffness shear-thinning alginate hydrogel	No drug cargo; designed as a catheter-deliverable intramyocardial mechanical support material	Percutaneous transendocardial intramyocardial injection using the dedicated EndoWings catheter system	First-in-human, single-arm Phase I study; NCT04781660	Patients with HFrEF, NYHA class III–IV, and LVEF ≤ 35%	Primary endpoint: procedure- or device-related serious adverse events within 30 days. Secondary endpoints included device success, heart failure hospitalization at 6 months, LVEF, NYHA class, 6-min walk distance, KCCQ score, and NT-proBNP	A 2025 report suggested preliminary safety and feasibility of percutaneous transendocardial alginate hydrogel injection in patients with HFrEF. Its translational significance lies in moving alginate-based myocardial injection from surgical delivery toward a less invasive catheter-based strategy	NCT04781660

## Biomaterial regulation of macrophages to promote inflammation resolution and repair

**Fig. 2:**
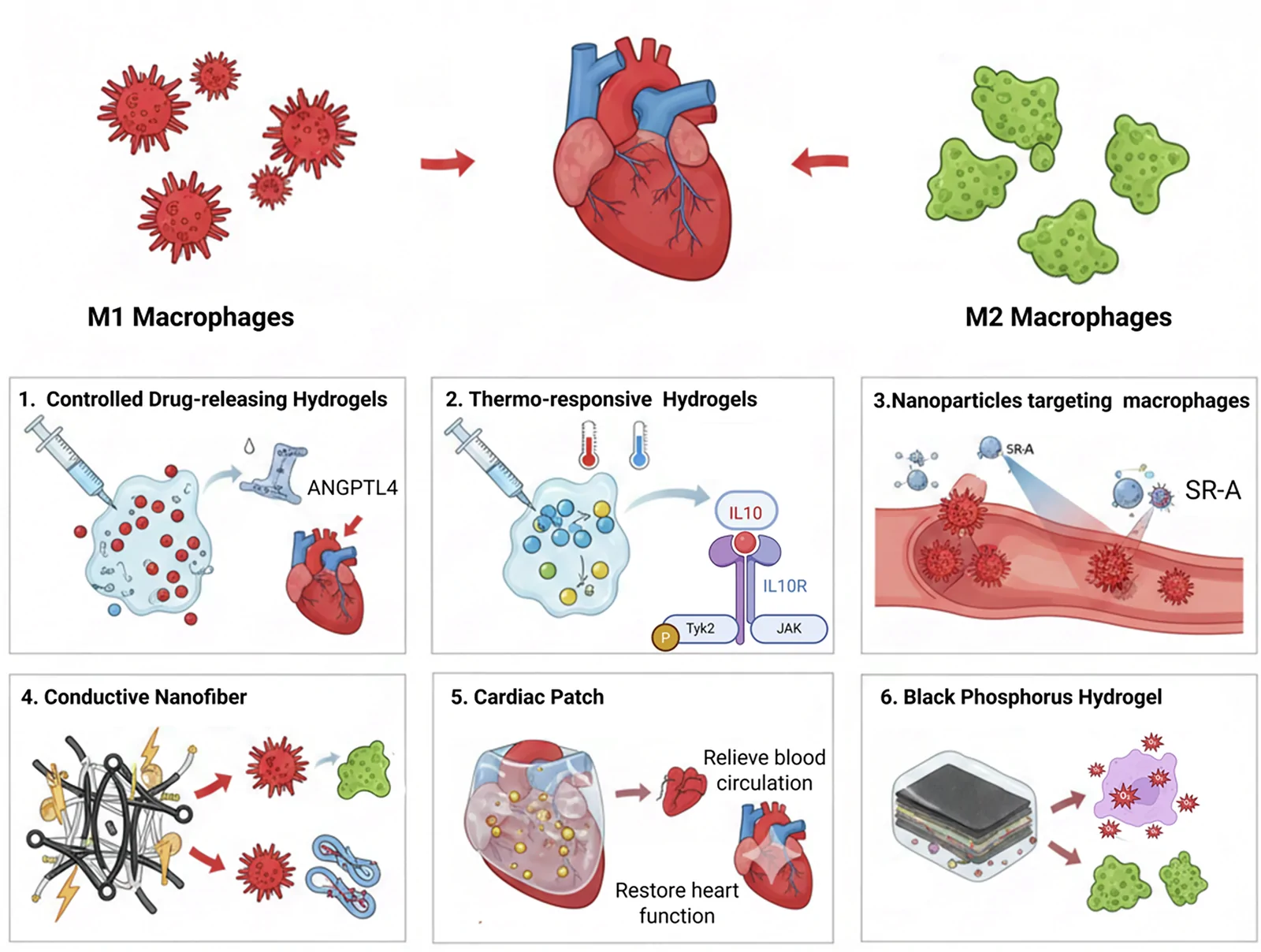
Biological materials regulate macrophages for the treatment of myocardial infarction

Macrophages are central determinants of post-MI healing, broadly transitioning from an early pro-inflammatory state to a reparative phenotype that supports angiogenesis, matrix deposition, and resolution [52]. Biomaterials can reprogram macrophage behavior through controlled therapeutic release, immuno-instructive chemistry, antioxidant activity, and electroactive interfaces (Fig. 2).

### Controllable drug-release hydrogels

Injectable hydrogels constructed from biomacromolecules (e.g., gelatin-based networks with aldehyde-functional polysaccharides) can provide sustained local release of anti-inflammatory proteins or immunomodulatory cues [53]. By prolonging residence time within the infarct, these systems can reduce pro-inflammatory cell infiltration and bias macrophage polarization toward reparative programs, while limiting systemic exposure.

### Thermoresponsive injectable gels for sequential delivery

Thermoresponsive matrices can enable staged release of proteins (e.g., early VEGF followed by IL-10), matching the physiological need for angiogenic initiation and subsequent inflammation resolution. Such sequential delivery can reduce excessive immune infiltration [53], enhance M2-like polarization, and improve functional indices in experimental MI models.

### Nanoparticles targeting inflammatory macrophages

Nanoparticle platforms can be engineered to bind macrophage scavenger receptors and deliver therapeutics to activated macrophages within atherosclerotic plaques or infarcted tissue [54]. When combined with externally triggerable modalities (e.g., low-intensity focused ultrasound), such systems can modulate macrophage burden or phenotype, suppor plaque stabilization or controlled inflammatory attenuation.

### Conductive nanofiber cardiac patches

Conductive composites incorporating nanocarbons or other conductive fillers can improve mechanical integrity and electrical coupling while shaping immune responses at the biomaterial–tissue interface [55]. These patches may promote reparative macrophage polarization, reduce scar formation, and support myocardial functional recovery.

### Antioxidant black phosphorus–based hydrogels

By scavenging ROS, antioxidant hydrogels can mitigate oxidative stress that sustains pro-inflammatory signaling [56]. Reduced oxidative burden can facilitate macrophage polarization toward reparative phenotypes and improve tissue preservation, offering a mechanistically coherent route to microenvironment normalization.

### Injectable matrices integrating drugs and ECM-mimetic cues

Hybrid injectable hydrogels co-delivering small molecules (e.g., flavonoids) and nanocomponents (e.g., silica nanoparticles) can combine anti-inflammatory [57], pro-angiogenic, and matrix-supportive functions. Such systems aim to suppress pathological inflammation while establishing permissive conditions for vascular ingrowth and reparative remodeling.

### Nanoengineered conductive collagen-based patch

Conductive collagen-based constructs incorporating nanoscale conductive elements (e.g., gold nanostructures) may enhance connexin-43 expression and electrical connectivity [58], while simultaneously presenting ECM cues that support cell survival and organized repair.

## Drug delivery biomaterials for the treatment of myocardial infarction

**Fig. 3:**
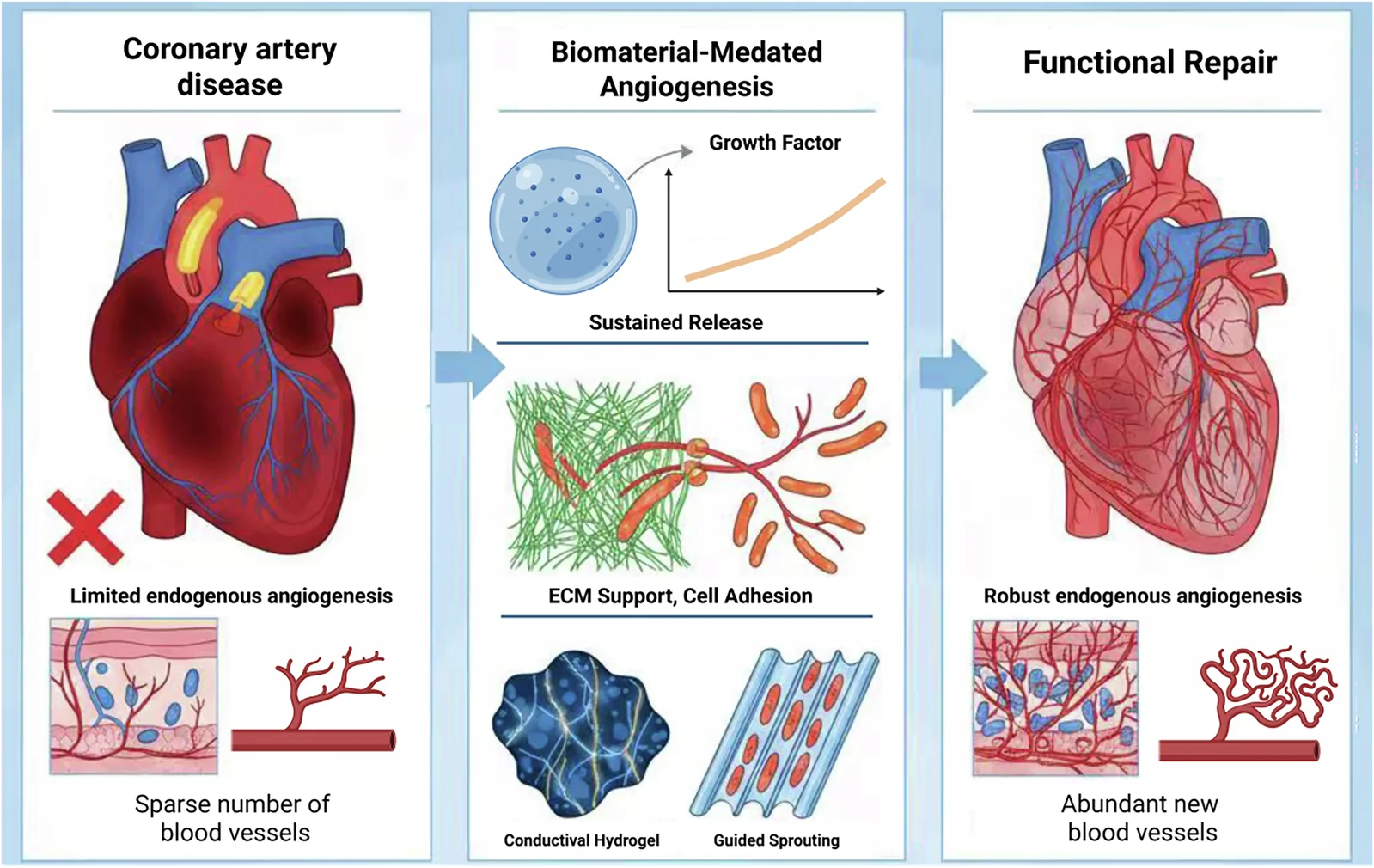
Biological materials regulate vascular regeneration for the treatment of myocardial infarction

In the field of myocardial infarction, or MI, the core rationale for biomaterial-based drug delivery is not simply “getting the drug to the heart.” Rather, it is to create local retention in the infarcted region, enable sustained release, modulate the microenvironment, and provide mechanical support, thereby influencing post-infarction inflammation, oxidative stress, angiogenesis, fibrosis, and ventricular remodeling (Fig. 3).

First, small-molecule drugs include antioxidants, anti-inflammatory agents, antiapoptotic agents, antifibrotic compounds, and mitochondrial protectants [59]. Representative examples include ROS scavengers, NF-κB inhibitors, MMP modulators, and antifibrotic agents. Biomaterials enhance their local concentration, reduce systemic side effects, and prolong their duration of action. Next, proteins and growth factors commonly include VEGF, bFGF, IGF-1, HGF, SDF-1α, and PDGF-BB [60]. They mainly promote angiogenesis, cell survival, progenitor cell recruitment, and tissue repair. Because these proteins have short half-lives, diffuse readily, and are prone to inactivation, hydrogels or micro- and nanoscale carriers are used to protect them and enable controlled release. Nucleic acid therapeutics include miRNA [61], siRNA, mRNA, and plasmid DNA. Examples include delivery of miR-21 and miR-126 to promote angiogenesis or reduce apoptosis, miR-29b to suppress fibrosis, siRNA to silence pro-inflammatory or profibrotic targets, and mRNA to express regenerative or pro-angiogenic factors. Extracellular vesicles [62], including exosomes, carry miRNAs, proteins, and lipids, and exert immunomodulatory, pro-angiogenic, and cardioprotective effects. Biomaterials improve their cardiac retention and reduce rapid clearance by the liver and spleen. Finally, cells or cell-derived factors such as MSCs, iPSC-derived cardiomyocytes, and endothelial progenitors have been explored for cardiac repair, but they often suffer from poor survival and retention. As a result, current strategies increasingly favor delivering the cell secretome, extracellular vesicles, or paracrine factors using biomaterial platforms, thereby reducing uncertainties associated with direct cell transplantation.

## Biomaterial strategies to regulate endothelial cells and promote vascular regeneration

Revascularization of the infarct and border zones is a prerequisite for durable myocardial repair, as it restores oxygen and nutrient delivery, removes metabolic waste, and supports survival and integration of resident and transplanted cells [63]. In adult hearts with limited cardiomyocyte regeneration, angiogenesis—sprouting from pre-existing vessels—represents a primary mechanism of post-infarction revascularization [64]. Insufficient vascular regeneration sustains hypoxia, accelerates remodeling, and compromises the efficacy of implanted cells or materials.

Traditional bolus delivery of angiogenic proteins (e.g., VEGF, bFGF) is limited by short half-life, rapid clearance, proteolysis, and poor tissue retention, often requiring high doses that may increase vascular leakiness or other adverse effects [65]. Biomaterials overcome these constraints by acting as “smart depots” that protect bioactivity and enable controlled, localized release to approximate physiological signaling gradients.

Polysaccharide-based hydrogels exemplify such platforms due to their ECM-mimetic hydration, tunable degradation, and tissue compatibility. Chitosan-based hydrogels can support vascular smooth muscle adhesion and, when loaded with VEGF/bFGF, enhance vessel caliber and density [66]. Calcium-crosslinked alginate hydrogels can physically encapsulate VEGF for sustained release, reducing burst-associated vascular leakage and aberrant angiogenesis. Protease-responsive systems further enable on-demand release tuned to the infarct microenvironment, where elevated protease activity can trigger liberation of tethered growth factors while concurrently limiting excessive ECM degradation [67].

In addition to biochemical delivery, biomaterials can incorporate mechanical and electrical functionalities to optimize vascular regeneration. Conductive hydrogels may indirectly enhance endothelial migration and sprouting by improving electromechanical synchrony [68], whereas topographically patterned scaffolds can guide endothelial alignment and promote hierarchical vessel architecture [69]. Together, these approaches advance vascular therapy from passive factor supplementation toward active microenvironmental control via coordinated “material–cell–factor” interactions.

## Biomaterial strategies to promote cardiomyocyte regeneration

**Fig. 4:**
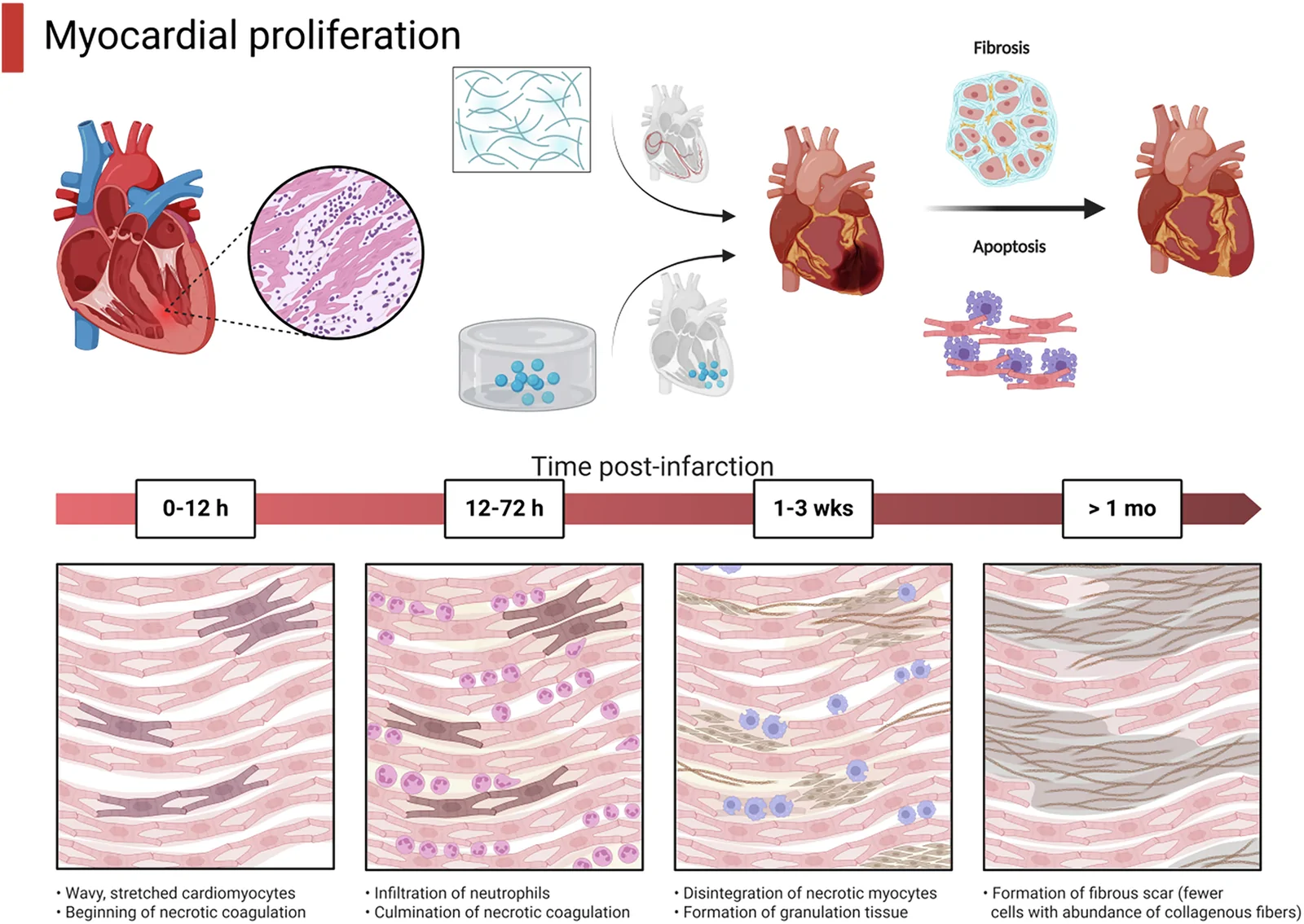
Biological materials regulate myocardial regeneration to treat myocardial infarction

Reconstituting cardiomyocyte mass is a central challenge in CAD and MI, as adult mammalian cardiomyocytes largely exit the cell cycle after birth. Loss of viable myocardium drives scar accumulation, chamber dilation, and eventual heart failure [70]. Consequently, reactivating cardiomyocyte proliferation—or enabling replacement through regenerative programs—has become a major research focus aimed at functional restoration rather than mere injury containment (Fig. 4).

Current pro-proliferative strategies often target metabolic reprogramming and cell-cycle regulatory axes. Mature cardiomyocytes preferentially rely on fatty acid oxidation, whereas proliferative neonatal-like states are more glycolytic [71]. Genetic or pharmacologic interventions that shift metabolic programs toward glycolysis, or modulate mitochondrial translation stress responses, have been shown to increase cardiomyocyte cell-cycle re-entry and reduce infarct burden in preclinical studies [72]. Complementary approaches overexpress cyclins and cyclin-dependent kinases (CDKs) to force cell-cycle activation, though they face challenges related to delivery efficiency, tissue specificity, and control of expression duration to minimize arrhythmogenic or oncogenic risks [73].

Biomaterials address these barriers through three principal mechanisms:

1. **Targeted delivery and controlled release of regenerative cues**.
  Hydrogels, nanoparticles, and scaffold systems can locally deliver nucleic acids (siRNA, mRNA, plasmids) [74, 75], gene-editing tools, or small molecules that activate cell-cycle or metabolic pathways, enabling sustained exposure in the infarct region while reducing off-target effects.
2. **Microenvironmental support for proliferation**.
  ECM-mimetic materials provide structural and biochemical support [76], while porous architectures facilitate nutrient diffusion and waste removal [77]. Aligned fibrous scaffolds can guide cardiomyocyte orientation, which is essential for tissue-level function.
3. **Multi-functional integration with immunomodulation and angiogenesis**.

Because inflammation, oxidative stress, and fibrosis constrain regeneration, biomaterials can co-deliver anti-inflammatory factors (e.g., IL-10) and pro-angiogenic signals (e.g., VEGF) alongside proliferative cues [78], enabling a permissive niche for cardiomyocyte renewal and maturation.

Despite progress, translation remains limited by low proliferative efficiency in adult myocardium and a hostile post-infarction milieu (oxidative stress, fibrosis, and electrical instability). Biomaterials provide a scalable framework for multi-dimensional regulation—combining delivery, niche engineering, and system-level coordination—to improve the safety and efficacy of cardiomyocyte regeneration strategies.

## Conclusion and perspective

Inflammation is not merely a bystander in CAD and MI but an organizing principle that governs plaque evolution, infarct injury, and post-MI remodeling [79]. Conventional therapies—while lifesaving in restoring perfusion—do not fully address the immune and ECM dysregulation that drives long-term functional decline. Biomaterial-based interventions provide a compelling, mechanistically grounded expansion of the therapeutic toolkit by enabling localized, time-programmed, and multi-modal microenvironment remodeling. Through controlled delivery of immunoregulatory and regenerative factors, mechanical stabilization of the infarct wall, electroactive coupling, and immuno-instructive interfaces that bias macrophage polarization, biomaterials can help break the pathological cycle of “injury → inflammation → remodeling.”

Looking forward, several directions are likely to accelerate both scientific impact and clinical translation [80]. Time-programmed (phase-adaptive) immunomodulation. Materials should be designed to align with the evolving needs of post-MI healing—early restraint of excessive inflammation, mid-phase support of angiogenesis and repair, and late limitation of maladaptive fibrosis. Stimuli-responsive platforms (ROS-, pH-, enzyme-, or mechanically activated systems) are particularly promising for achieving “on-demand” regulation. Precision targeting informed by single-cell and spatial omics. Single-cell transcriptomics and spatial profiling can resolve immune and stromal heterogeneity across infarct, border, and remote zones, enabling rational selection of targets (cytokines, receptors, ECM enzymes) and guiding region-specific delivery strategies. Multifunctional integration without compromising safety. Future constructs must co-optimize conductivity, mechanical compliance, biodegradability, and bioactivity, while maintaining predictable degradation kinetics and non-toxic byproducts.

Clinically compatible delivery and manufacturability. Minimally invasive administration (e.g., catheter-based injection, epicardial delivery with reduced invasiveness) and scalable manufacturing under quality-controlled conditions will be critical for translation. Rigorous evaluation of long-term outcomes. Beyond short-term infarct size reduction, studies should prioritize long-term LV function, arrhythmia risk, scar quality, vascular maturity, and systemic immune effects, together with standardized large-animal validation where appropriate. In summary, biomaterials that actively instruct immune and reparative programs have the potential to shift CAD/MI management from “reperfusion-first” toward microenvironment-guided functional reconstruction. Continued convergence of materials science, immunology, and systems-level profiling is expected to enable next-generation therapies that are both mechanistically precise and clinically deployable.

### BRIEFS

This review highlights immuno-instructive biomaterials as a complementary approach to reprogram the post-infarction microenvironment, restore immune homeostasis, and accelerate functional repair in CAD and MI by integrating spatiotemporally controlled immunomodulation, mechanical stabilization, and multifunctional design.

### SYNOPSIS

Coronary artery disease (CAD) and myocardial infarction (MI) evolve within dynamic inflammatory microenvironments that constrain the benefits of reperfusion, underscoring the need for strategies that correct immune dysregulation while promoting repair. This review summarizes recent advances in immuno-instructive biomaterials that enable localized, time-programmed therapeutic delivery, infarct-wall reinforcement, immune-cell re-education (notably macrophage polarization toward reparative states), and electro-mechanical integration to reshape the post-ischemic niche. We discuss major modalities including spatiotemporally controlled release of cytokines/growth factors, ECM-mimetic scaffolds for structural support, and conductive or antioxidant materials to coordinate immune regulation with regeneration. The review further examines microenvironmental remodeling through macrophage reprogramming, endothelial-driven neovascularization, and cardiomyocyte-protective/pro-proliferative cues, emphasizing phase-adaptive intervention (early inflammation containment, mid-phase repair amplification, late-stage fibrosis mitigation) and precision targeting guided by single-cell omics. Remaining translational barriers include delivery invasiveness, robust multifunctional integration, scalable manufacturing, and long-term safety, motivating the development of stimuli-responsive and clinically deployable platforms that move CAD/MI care beyond “reperfusion-first” toward microenvironment-guided functional reconstruction.

## Abbreviations

CAD: Coronary artery disease
ECM: Extracellular matrix
LV: left ventricular
MI: myocardial infarction
